# Measurement Model and Precision Analysis of Accelerometers for Maglev Vibration Isolation Platforms

**DOI:** 10.3390/s150820053

**Published:** 2015-08-14

**Authors:** Qianqian Wu, Honghao Yue, Rongqiang Liu, Xiaoyou Zhang, Liang Ding, Tian Liang, Zongquan Deng

**Affiliations:** 1 School of Mechatronics Engineering, Harbin Institute of Technology, Harbin 150001, China; E-Mails: liurq@hit.edu.cn (R.L.); liangding@hit.edu.cn (L.D.); dengzq@hit.edu.cn (Z.D.); 2 Department of Mechanical Engineering, Nippon Institute of Technology, Saitama 345-8501, Japan; E-Mail: zhang@nit.ac.jp; 3 School of Engine and Energy, Northwestern Polytechnical University, Xi’an 71000, China; E-Mail: snowandblue@126.com

**Keywords:** angular acceleration, measurement precision, configuration, root mean square noise

## Abstract

High precision measurement of acceleration levels is required to allow active control for vibration isolation platforms. It is necessary to propose an accelerometer configuration measurement model that yields such a high measuring precision. In this paper, an accelerometer configuration to improve measurement accuracy is proposed. The corresponding calculation formulas of the angular acceleration were derived through theoretical analysis. A method is presented to minimize angular acceleration noise based on analysis of the root mean square noise of the angular acceleration. Moreover, the influence of installation position errors and accelerometer orientation errors on the calculation precision of the angular acceleration is studied. Comparisons of the output differences between the proposed configuration and the previous planar triangle configuration under the same installation errors are conducted by simulation. The simulation results show that installation errors have a relatively small impact on the calculation accuracy of the proposed configuration. To further verify the high calculation precision of the proposed configuration, experiments are carried out for both the proposed configuration and the planar triangle configuration. On the basis of the results of simulations and experiments, it can be concluded that the proposed configuration has higher angular acceleration calculation precision and can be applied to different platforms.

## 1. Introduction

High-resolution satellites and scientific experiments in space demand high environment quality; however, micro-vibrations in the space environment seriously affects the accuracy of these scientific activities [1,2,3]. A maglev vibration isolation platform with six degrees of freedom (DOF), which has been successfully used in the field of low frequency vibration isolation, can significantly reduce the impact of micro-vibrations on experimental equipment and thereby increase the accuracy of scientific research [4,5,6].

**Figure 1:**
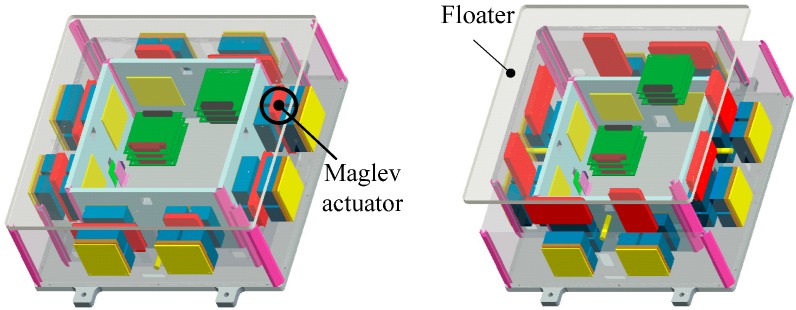
Schematic diagram of the active vibration isolation platform.

The maglev vibration isolation platform with six DOF is composed of a stator and a floater, as shown in Figure 1. The stator is fixed on the spacecraft, where various disturbances exist that are caused by power generation systems, maintenance systems, thermal control systems, *etc.* The floater is used to fix payloads, such as ultra-precise equipment, sensitive loads, high-resolution telescopes *etc.* [7,8]. In order to provide an acceptable acceleration level for the payload, sensing of the absolute acceleration of the vibration level is necessary. Appropriate control algorithms can be designed to produce the corresponding forces using maglev actuators to counteract the measured vibration [9,10]. As the acceleration magnitude of the disturbances is small in a micro-vibration environment, high measurement precision is especially important for the improvement of isolation performance. 

**Figure 2:**
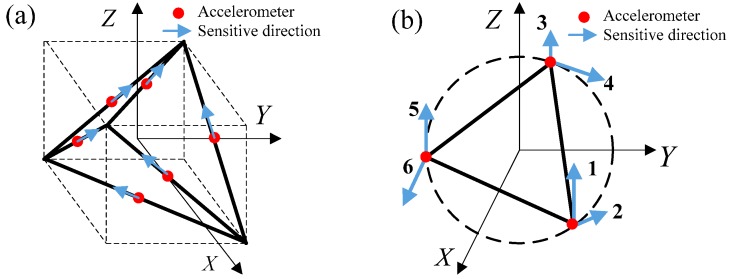
Two typical accelerometer configurations. (**a**) Cubic configuration; (**b**) planar triangle configuration.

The method used to measure angular acceleration by linear accelerometers was first proposed as early as 1965 [11]. The angular acceleration measurement precision depends closely on the accelerometer configuration. Two typical configurations that use the minimum number of accelerometers to measure six degrees of freedom accelerations have been developed in past research, as shown in Figure 2. A cubic configuration presented by Chen in 1994 [12] is shown in Figure 2a. Six accelerometers are fixed at the center of each cube’s six faces to detect six DOF motion, and the sensitive directions are set along the diagonal directions [12,13]. The method has been proven to have high accuracy and reliability in some studies [14]. However, the cubic configuration is suitable only when the space available is cubic and it requires high installation precision. In addition, a planar triangle configuration of the accelerometers to detect six DOF accelerations has been applied to an active vibration isolation platform [15]. As shown in Figure 2b, six accelerometers are fixed on three vertices of an equilateral triangle. Three of the sensitive directions are vertical. Others are along the tangential direction of a circumcircle of the equilateral triangle. Even on the same plane, the planar triangle configuration needs three vertexes of an equilateral triangle. In an electrical system, the appropriate space is rare and the sensitive directions need high installation precision.

The aim of this work is to propose a measurement model that uses a minimum number of accelerometers but has high measurement precision for six DOF accelerations. In order to overcome the shortcomings of existing configurations mentioned above, such as space constraints, installation precision demands, and so on, a new accelerometer configuration for measuring six DOF accelerations was proposed in this paper. The conditions needed for minimizing angular acceleration calculation noise are presented. Moreover, a comparison was carried out between the proposed configuration and the planar triangle configuration. The effect of the installation position and orientation errors on the output values of accelerometers for these two configurations were analyzed by simulation. The calculation precision of angular acceleration was compared for the two configurations by practical experiments.

## 2. Analysis of Angular Acceleration Calculation

**Figure 3:**
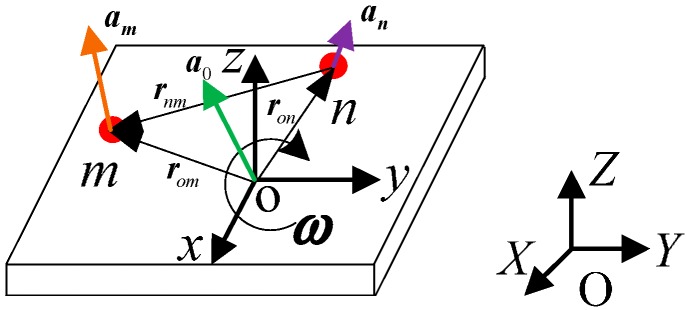
Schematic diagram of a rigid body.

As shown in Figure 3, O-XYZ is the inertial frame and o-xyz is the body frame. Point *m* and point *n* are selected arbitrarily on a rigid body. According to the dynamics theory of rigid bodies, the acceleration of any point on a rigid body contains inertial acceleration, centripetal acceleration and tangential acceleration.

The accelerations of point *m* and point *n* in the inertial frame can be expressed by Equations (1) and (2), respectively:

(1) $$a_{m}=a_{o}+\alpha \times r_{om}+\omega \times \left(\omega \times r_{om}\right)$$

(2) $$a_{n}=a_{o}+\alpha \times r_{on}+\omega \times \left(\omega \times r_{on}\right)$$

where $a_{o}$ is the inertial acceleration of the coordinate origin; $\alpha ={\left[\begin{array}{ccc} \alpha_{x} & \alpha_{y} & \alpha_{z} \end{array}\right]}^{T}$ is the angular acceleration vector relative to the body frame; $r_{om}$ and $r_{on}$ are the displacement vector from the coordinate origin to the point *m* and *n*, respectively; $\omega ={\left[\begin{array}{ccc} \omega_{x} & \omega_{y} & \omega_{z} \end{array}\right]}^{T}$ is the angular velocity relative to the body frame.

The difference of the acceleration between *m* and *n* can be written as Equation (3):

(3) $$a_{mn}=a_{m}-a_{n}=\alpha \times r_{nm}+\omega \times \left(\omega \times r_{nm}\right)$$

where $r_{nm}={\left[\begin{array}{ccc} r_{nmx} & r_{nmy} & r_{nmz} \end{array}\right]}^{T}$ is the displacement vector from point *m* to point *n*.

The matrix form of Equation (3) is written as follows:

(4) $$\left[\begin{array}{c} a_{mnx}\\ a_{mny}\\ a_{mnz} \end{array}\right]=\left[\begin{array}{c} \alpha_{x}\\ \alpha_{y}\\ \alpha_{z} \end{array}\right]\times \left[\begin{array}{c} r_{nmx}\\ r_{nmy}\\ r_{nmz} \end{array}\right]+\left[\begin{array}{c} \omega_{x}\\ \omega_{y}\\ \omega_{z} \end{array}\right]\times \left(\left[\begin{array}{c} \omega_{x}\\ \omega_{y}\\ \omega_{z} \end{array}\right]\times \left[\begin{array}{c} r_{nmx}\\ r_{nmy}\\ r_{nmz} \end{array}\right]\right)$$

where each item in the equations contains plus or minus. Equation (4) can be solved as:

(5) $$\left[\begin{array}{c} a_{mnx}\\ a_{mny}\\ a_{mnz} \end{array}\right]=\left[\begin{array}{c} \alpha_{y}r_{nmz}-\alpha_{z}r_{nmy}\\ -\alpha_{x}r_{nmz}+\alpha_{z}r_{nmx}\\ \alpha_{x}r_{nmy}-\alpha_{y}r_{nmx} \end{array}\right]+\left[\begin{array}{c} \omega_{x}\omega_{y}r_{nmy}-\omega_{y}\omega_{y}r_{nmx}+\omega_{x}\omega_{y}r_{nmz}-\omega_{x}\omega_{y}r_{nmx}\\ -\omega_{x}\omega_{x}r_{nmy}+\omega_{y}\omega_{y}r_{nmx}+\omega_{y}\omega_{z}r_{nmz}-\omega_{z}\omega_{z}r_{nmy}\\ -\omega_{x}\omega_{y}r_{nmz}+\omega_{x}\omega_{z}r_{nmx}-\omega_{y}\omega_{y}r_{nmz}+\omega_{y}\omega_{z}r_{nmy} \end{array}\right]$$

As the magnitude of micro-vibrations is tiny, almost all the research about maglev vibration isolation platforms is only focused on the acceleration level [3,9,16]. The squared terms and cross product terms of the angular velocity are all neglected to construct a dynamic model of the system with high precision [17,18]. This can be done in order to simplify theoretical analysis for the section.

Therefore, Equation (5) can be simplified as:

(6) $$\left[\begin{array}{c} a_{mnx}\\ a_{mny}\\ a_{mnz} \end{array}\right]=\left[\begin{array}{c} \alpha_{y}r_{nmz}-\alpha_{z}r_{nmy}\\ -\alpha_{x}r_{nmz}+\alpha_{z}r_{nmx}\\ \alpha_{x}r_{nmy}-\alpha_{y}r_{nmx} \end{array}\right]$$

If an accelerometer ***A****_i_* is installed at the point $r_{i}$ on the rigid body, and the sensitive direction cosine of the accelerometer is $\theta_{i}$, then the output value of the accelerometer can be written as:

(7) $$a_{i}=\left[a_{o}+\alpha \times r_{i}+\omega \times \left(\omega \times r_{i}\right)\right]\cdot \theta_{i}$$

### 2.1. Configuration for One DOF Angular Acceleration

**Figure 4:**
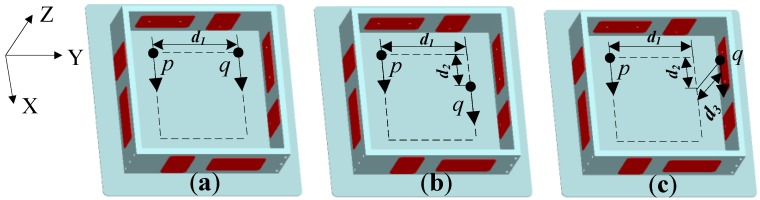
Configurations for detecting one DOF angular acceleration.

Taking the floater as an example, three placements listed in Figure 4 can be used to acquire one DOF angular acceleration of a rigid body. The sensitive directions of accelerometers *p* and *q* are assumed to be along the X axis. The distances between *p* and *q* along X, Y and Z directions are *d*_2_, *d*_1_, and *d*_3_, respectively. Then, the angular acceleration could be calculated based on output values of the accelerometers.

As the X axis is the sensitive direction, only the acceleration along the X direction was considered. According to Equation (6), the difference of output values between accelerometers *p* and *q* in Figure 4a,b can be deduced as:

(8) $$a_{pqx}=a_{px}-a_{qx}=\alpha_{y}r_{qpz}-\alpha_{z}r_{qpy}=\alpha_{z}d_{1}$$

According to Equation (8), angular acceleration around the Z axis in Figure 4a,b can be obtained:

(9) $$\alpha_{z}=\frac{a_{px}-a_{qx}}{d_{1}}$$

Based on Figure 4c, the difference of output values between accelerometers *p* and *q* is:

(10) $$a_{pqx}=\alpha_{y}r_{qpz}-\alpha_{z}r_{qpy}=-\alpha_{y}d_{3}+\alpha_{z}d_{1}$$

Thus, the angular acceleration around the Z axis in Figure 4c is:

(11) $$\alpha_{z}=\frac{a_{px}-a_{qx}}{d_{1}}-\frac{\alpha_{y}d_{3}}{d_{1}}$$

Equation (11) shows that the angular acceleration around the Y axis must be known first to calculate the angular acceleration around the Z axis. The distance *d*_3_ will bring more noise to the angular acceleration. Therefore, Figure 4c is inadvisable, so it can be seen that a coplanar accelerometer configuration has higher measurement precision than a non-coplanar placement and the accelerometers can be placed on any point of a plane to obtain one DOF angular acceleration with high precision.

### 2.2. Configuration for Two DOF Angular Acceleration

**Figure 5:**
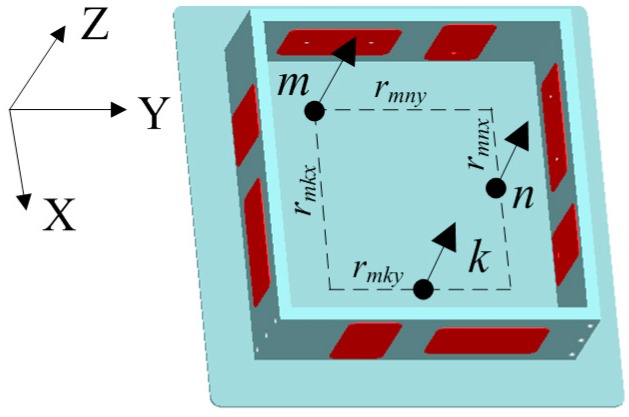
Configuration for detecting two DOF angular acceleration.

The above analysis indicates that four accelerometers can measure two DOF angular accelerations. To save space and decrease cost, three accelerometers are enough to detect two DOF angular accelerations by sharing an accelerometer with another two accelerometers. As shown in Figure 5, three accelerometers *m*, *n*, *k* are placed on the same plane and the sensitive directions are all along the Z axis.

According to Equation (6):

(12) $$a_{mnz}=a_{mz}-a_{nz}=\alpha_{x}r_{nmy}-\alpha_{y}r_{nmx}$$

(13) $$a_{mkz}=a_{mz}-a_{kz}=\alpha_{x}r_{kmy}-\alpha_{y}r_{kmx}$$

Solving Equations (12) and (13), the angular accelerations around the X axis and Y axis are:

(14) $$\alpha_{x}=\frac{r_{kmx}\left(a_{mz}-a_{nz}\right)-r_{nmx}\left(a_{mz}-a_{kz}\right)}{r_{nmy}r_{kmx}-r_{kmy}r_{nmx}}$$

(15) $$\alpha_{y}=\frac{r_{kmy}\left(a_{mz}-a_{nz}\right)-r_{nmy}\left(a_{mz}-a_{kz}\right)}{r_{nmy}r_{kmx}-r_{kmy}r_{nmx}}$$

### 2.3. Configuration for Six DOF Angular Acceleration

**Figure 6:**
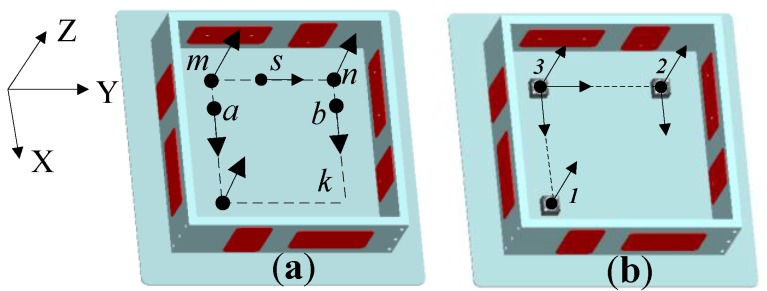
Configuration of accelerometers for a floater.

Based on the previous analysis, at least six accelerometers are needed to measure six DOF accelerations of a platform. As shown in Figure 6a, by adopting six single-axis accelerometers *m*, *n*, *k*, *p*, *q* and *s*, three linear accelerations and three angular accelerations of the floater can be calculated.

In general, multi-axis integrated accelerometers are used to save space, which not only improves the installation accuracy, but also reduces cost. The floater is taken as an example. A three-axis integrated accelerometer, a two-axis integrated accelerometer and a single-axis accelerometer are adopted. The configuration of the accelerometers after assembly is displayed in Figure 6b. Based on Equation (7), the output of each accelerometer can be calculated:

(16) $$\left\{ \begin{array}{l} a_{1z}=a_{z}-r_{23y}\alpha_{x}/2-r_{21x}\alpha_{y}/2\\ a_{2x}=a_{x}-r_{21y}\alpha_{z}/2\\ a_{2z}=a_{z}+r_{23y}\alpha_{x}/2+r_{21x}\alpha_{y}/2\\ a_{3x}=a_{x}+r_{21y}\alpha_{z}/2\\ a_{3y}=a_{y}-r_{21x}\alpha_{z}/2\\ a_{3z}=a_{z}-r_{23y}\alpha_{x}/2+r_{21x}\alpha_{y}/2 \end{array}\right.$$

Assuming the distances between any two accelerometers space can be represented by *d* because all sides are equal, then the linear and the angular accelerations of the floater can be solved and simplified as Equation (17):

(17) $$\left\{ \begin{array}{l} a_{x}=\left(a_{3x}+a_{2x}\right)/2\\ a_{y}=\left(2a_{3y}+a_{3x}-a_{2x}\right)/2\\ a_{z}=\left(a_{2z}+a_{1z}\right)/2\\ \alpha_{x}=\left(a_{2z}-a_{3z}\right)/d\\ \alpha_{y}=\left(a_{3z}-a_{1z}\right)/d\\ \alpha_{z}=\left(a_{3x}-a_{2x}\right)/d \end{array}\right.$$

Based on the theory of random variables, the root mean square (RMS) noise of the angular acceleration can be calculated as follows:

(18) $$\sigma_{\alpha }=\sqrt{\sigma_{\alpha_{x}}^{2}+\sigma_{\alpha_{y}}^{2}+\sigma_{\alpha_{y}}^{2}}$$

where $\sigma_{\alpha_{x}}$, $\sigma_{\alpha_{y}}$ and $\sigma_{\alpha_{z}}$ are the RMS noise of angular acceleration about X axis, Y axis and Z axis, respectively. 

According to Equation (17), RMS noise of each angular acceleration can be calculated by Equation (19):

(19) $$\begin{array}{l} \sigma_{\alpha_{x}}=\frac{\sqrt{\sigma_{a_{2z}}^{2}+\sigma_{a_{3z}}^{2}}}{d}\\ \sigma_{\alpha_{y}}=\frac{\sqrt{\sigma_{a_{2z}}^{2}+\sigma_{a_{1z}}^{2}}}{d}\\ \sigma_{\alpha_{x}}=\frac{\sqrt{\sigma_{a_{3x}}^{2}+\sigma_{a_{2x}}^{2}}}{d} \end{array}$$

where $\sigma_{a_{2x}}$, $\sigma_{a_{3x}}$, $\sigma_{a_{1z}}$, $\sigma_{a_{2z}}$, $\sigma_{a_{3z}}$ are the variances of noise outputs of each accelerometer.

According to Equation (19), maximizing the distance *d* as much as possible in the effective installation space is the best way to minimize the calculation noise of angular acceleration and improve measurement precision.

For the planar triangle configuration, the outputs of each accelerometer are assumed to be *a*_1_~*a*_6_ and the radius of the circumcircle is *R*. Then linear acceleration and angular acceleration can be calculated by Equation (20) [15]. Similarly, the noise of the angular acceleration decreases with increasing radius *R*:

(20) $$\left\{ \begin{array}{l} a_{x}=\left(2a_{6}-a_{2}-a_{4}\right)/3\\ a_{y}=\sqrt{3}\left(a_{2}-a_{4}\right)/3\\ a_{z}=\left(a_{1}+a_{3}+a_{5}\right)/3\\ \alpha_{x}=\left(a_{1}+a_{3}-2a_{5}\right)/(3R)\\ \alpha_{y}=\sqrt{3}\left(a_{3}-a_{1}\right)/(3R)\\ \alpha_{z}=\left(a_{2}\text{+}a_{4}+a_{6}\right)/(3R) \end{array}\right.$$

## 3. Simulations of the Effect of Installation Error on the Output Values of Accelerometers

Sensor inaccuracies, such as installation error, base bending, temperature transients, cable whip and so on, will deviate the output electric signal of the accelerometer from what it would have been, but inaccuracies such as base bending, temperature transients and cable whip, are hard to describe for the analysis. As the same accelerometers and the same installation plane are assumed for comparison in the same environment, the influence of base bending, temperature transients and cable whip can be neglected for the simulation analysis. Only the installation position error and orientation error are mainly considered for the simulation. The installation error is composed of the installation position error and sensitive direction error. Position installation error refers to a small displacement that deviates from the ideal installation position. The sensitive direction error is generally caused by a small rotation angle rotating around the ideal sensitive direction.

In this paper, the accelerometers are placed on a plane. The ideal installation position is:

(21) $$r=\frac{1}{2}\left[\begin{array}{cccccc} d & -d & -d & -d & -d & -d\\ -d & d & d & -d & -d & -d\\ 0 & 0 & 0 & 0 & 0 & 0 \end{array}\right]$$

The ideal sensitive direction is:

(22) $$\theta =\left[\begin{array}{cccccc} 0 & 1 & 0 & 1 & 0 & 0\\ 0 & 0 & 0 & 0 & 1 & 0\\ 1 & 0 & 1 & 0 & 0 & 1 \end{array}\right]$$

Assuming position installation errors are $\Delta r_{xi}$, $\Delta r_{yi}$ and $\Delta r_{zi}$ (*i* = 1, 2, 3), respectively, then the actual installation position is:

(23) $$r\prime =\left[\begin{array}{cccccc} \frac{d}{2}+\Delta r_{x1} & -\frac{d}{2}+\Delta r_{x2} & -\frac{d}{2}+\Delta r_{x2} & -\frac{d}{2}+\Delta r_{x3} & -\frac{d}{2}+\Delta r_{x3} & -\frac{d}{2}+\Delta r_{x3}\\ -\frac{d}{2}+\Delta r_{y1} & \frac{d}{2}+\Delta r_{y2} & \frac{d}{2}+\Delta r_{y2} & \frac{d}{2}+\Delta r_{y3} & -\frac{d}{2}+\Delta r_{y3} & -\frac{d}{2}+\Delta r_{y3}\\ \Delta r_{z1} & \Delta r_{z2} & \Delta r_{z2} & \Delta r_{z3} & \Delta r_{z3} & \Delta r_{z3} \end{array}\right]$$

**Figure 7:**
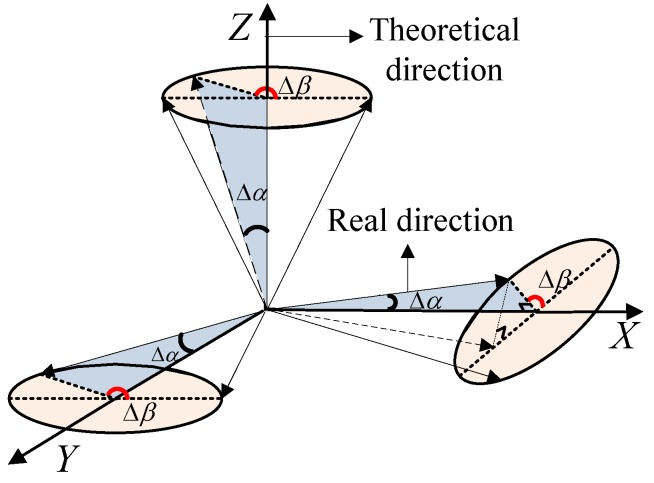
Sensitive direction error for a three-axis accelerometer.

For the maglev vibration isolation platform, the order of magnitude of the angular error Δ*α* is about 0.01°. With a three-axis accelerometer taken as a case, as shown in Figure 7, the theoretical directions are the X-axis, Y-axis and Z-axis, respectively. The angular offset is assumed to be Δα as the blue shading in the figure. The direction of the angular error is defined as Δβ, which can be any direction around the sensitive axis in the range of 0° to 360°. The actual sensitive directions are:

$$\begin{array}{l} \theta_{RX}={\left[\begin{array}{ccc} \cos\left(\Delta \alpha \right) & \sin\left(\Delta \alpha \right)\cos\left(\Delta \beta \right) & \sin\left(\Delta \alpha \right)\sin\left(\Delta \beta \right) \end{array}\right]}^{T}\\ \theta_{RY}={\left[\begin{array}{ccc} \sin\left(\Delta \alpha \right)\cos\left(\Delta \beta \right) & \cos\left(\Delta \alpha \right) & \sin\left(\Delta \alpha \right)\sin\left(\Delta \beta \right) \end{array}\right]}^{T}\\ \theta_{RZ}={\left[\begin{array}{ccc} \sin\left(\Delta \alpha \right)\cos\left(\Delta \beta \right) & \sin\left(\Delta \alpha \right)\sin\left(\Delta \beta \right) & \cos\left(\Delta \alpha \right) \end{array}\right]}^{T} \end{array}$$

Therefore, the actual sensitive direction cosine of the proposed configuration yields:

(24) $$\theta \prime =\left[\begin{array}{cccccc} \sin\left(\Delta \alpha \right)\cos\left(\Delta \beta \right) & \cos\left(\Delta \alpha \right) & \sin\left(\Delta \alpha \right)\cos\left(\Delta \beta \right) & \cos\left(\Delta \alpha \right) & \sin\left(\Delta \alpha \right)\cos\left(\Delta \beta \right) & \sin\left(\Delta \alpha \right)\cos\left(\Delta \beta \right)\\ \sin\left(\Delta \alpha \right)\sin\left(\Delta \beta \right) & \sin\left(\Delta \alpha \right)\cos\left(\Delta \beta \right) & \sin\left(\Delta \alpha \right)\sin\left(\Delta \beta \right) & \sin\left(\Delta \alpha \right)\cos\left(\Delta \beta \right) & \cos\left(\Delta \alpha \right) & \sin\left(\Delta \alpha \right)\sin\left(\Delta \beta \right)\\ \cos\left(\Delta \alpha \right) & \sin\left(\Delta \alpha \right)\sin\left(\Delta \beta \right) & \cos\left(\Delta \alpha \right) & \sin\left(\Delta \alpha \right)\sin\left(\Delta \beta \right) & \sin\left(\Delta \alpha \right)\sin\left(\Delta \beta \right) & \cos\left(\Delta \alpha \right) \end{array}\right]$$

Then, according to Equation (7), the linear acceleration and angular acceleration can be obtained.

**Figure 8:**
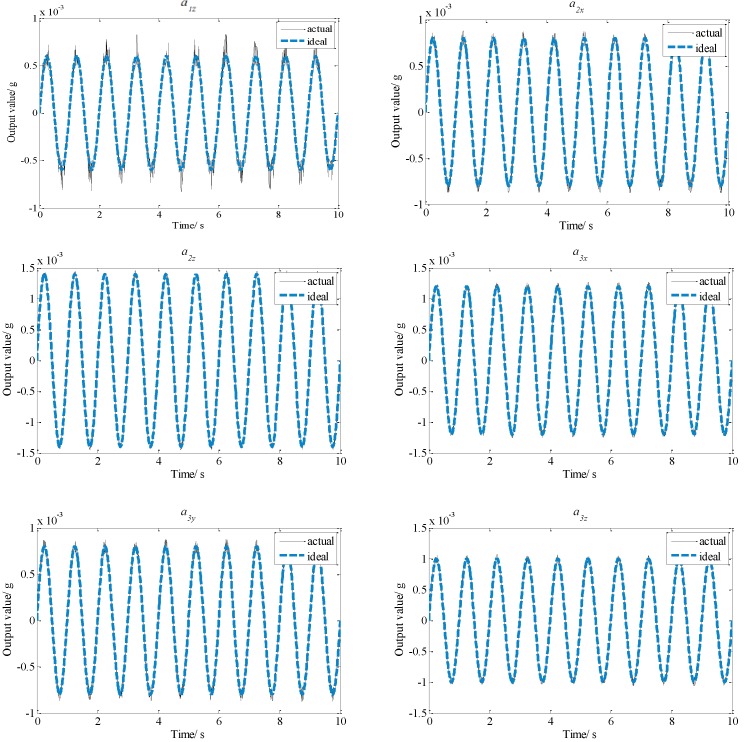
Comparison of output results of the proposed configuration.

To acquire the influence of the installation errors on the output value of each accelerometer, simulations were conducted. It is assumed that the linear accelerations and the angular accelerations of the floater are all sinusoidal motions with 1 Hz frequency and 1 micro-g amplitude. The distance *d* is about 0.4 m for the platform. The position installation errors $\Delta r_{x}$, $\Delta r_{y}$ and $\Delta r_{z}$ (*i* = 1, 2, 3) are all 0.0005 m, and the magnitude of the angular error Δ*α* is 0.05°. According to Equation (7) please confirm here is Equation (7), comparison results of output values between the proposed configurations with and without installation error are shown in Figure 8.

**Figure 9:**
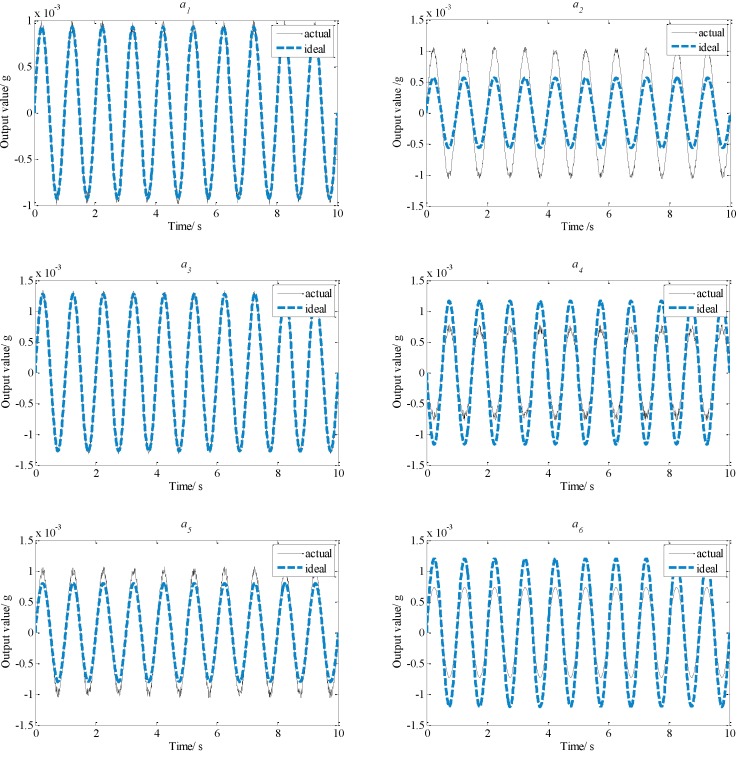
Comparison of output results of planar triangle configuration.

According to the planar triangle configuration, the comparison between the output values of the ideal installation and the actual installation under the same assumptions are displayed in Figure 9.

From Figure 8, it can be seen that actual output values of other accelerometers are almost the same as ideal output values. However, Figure 9 shows that the differences of most output values between the ideal values and the actual values of the planar triangle configuration are significant, so the total influence of the installation error on the output values of the proposed configuration is smaller than that on the planar triangle configuration. That indicates that the proposed configuration has higher measurement precision under the condition of identical installation errors. 

## 4. Experiments

**Figure 10:**
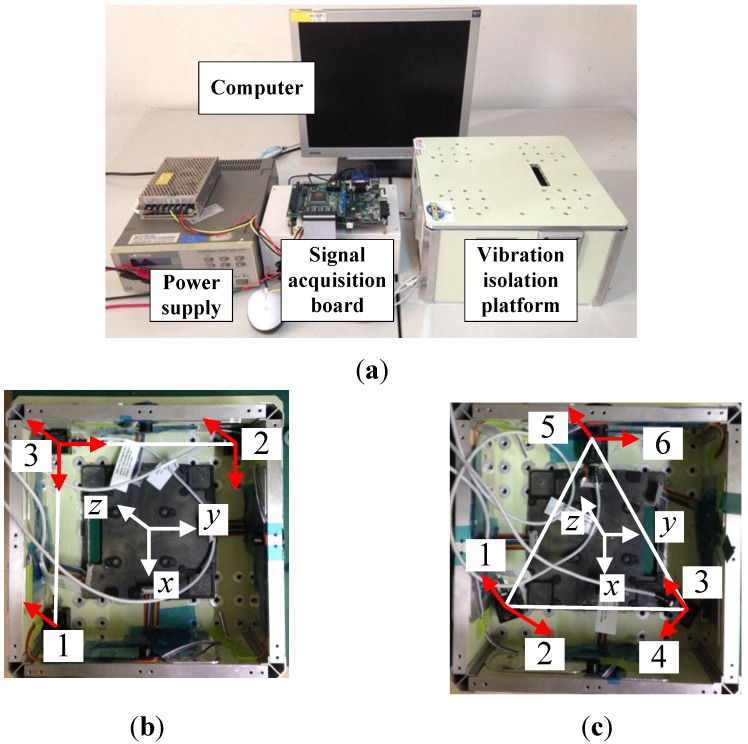
Experimental setups. (**a**) Signal measurement and processing system; (**b**) The proposed configuration; (**c**) The planar triangle configuration.

Experiments are carried out to verify the advantages of the proposed configuration by comparing angular accelerations obtained from the proposed configuration and the planar triangle configuration. Experimental setups are shown in Figure 10. Model 2422 accelerometers from Silicon were adopted to test the acceleration. The error is about 12 μg/(root Hz), the sensitivity is 2 V/g and its range is ±2 g. Only the reduction of the RMS error is considered and compared, as different vibrations exist in the environment during the experiments. Two accelerometer configurations were installed on the floater of the vibration isolation system and the output value of each accelerometer was acquired. First, the whole experimental setup is stationary. As there are many kinds of disturbances presented in the environment, the output values of the accelerometers contain environment noise and system noise. The RMS noises of angular accelerations of the two configurations are listed in Table 1. The data shows that the RMS noise of the angular acceleration of the proposed configuration has a 14.89% reduction compared to that of the planar triangle configuration in the same environment.

**Table 1:** Comparison of root mean square (RMS) noise of angular acceleration.

RMS Noise	The Proposed Configuration	The Planar Triangle Configuration
σ_α__x_	0.0117	0.0142
σ_α__y_	0.0119	0.0152
σ_α__z_	0.0118	0.0120
Noise reduction	14.89%	0

**Figure 11:**
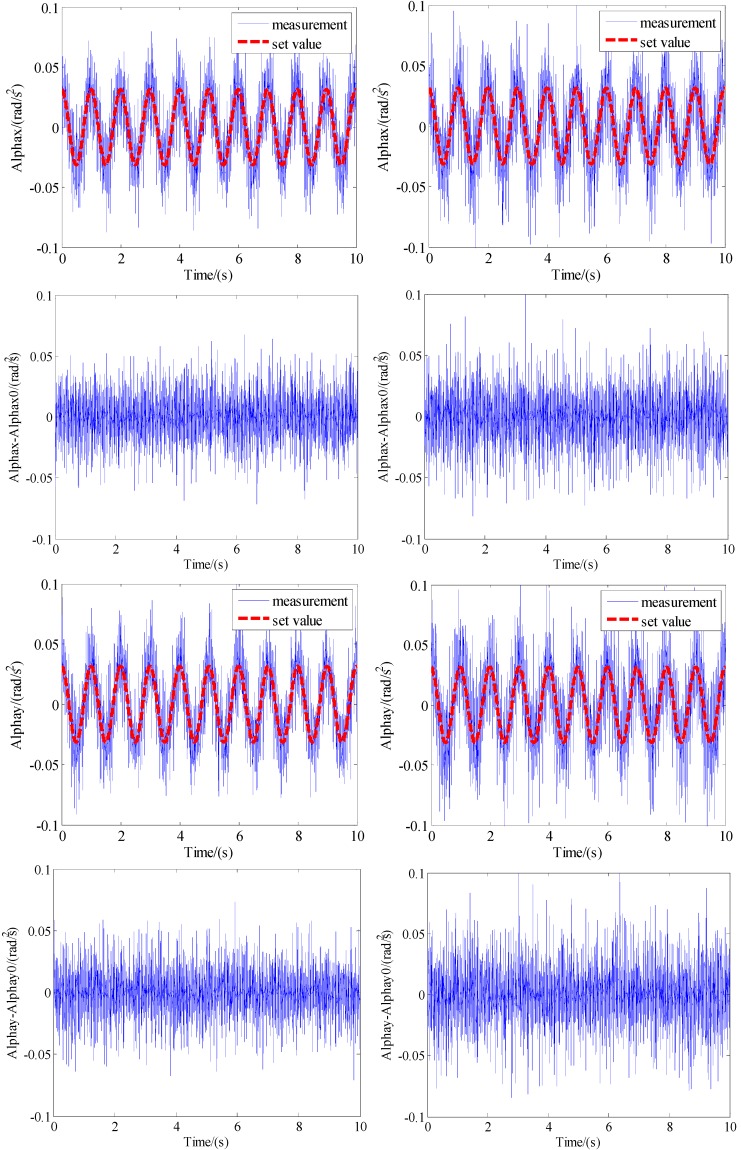

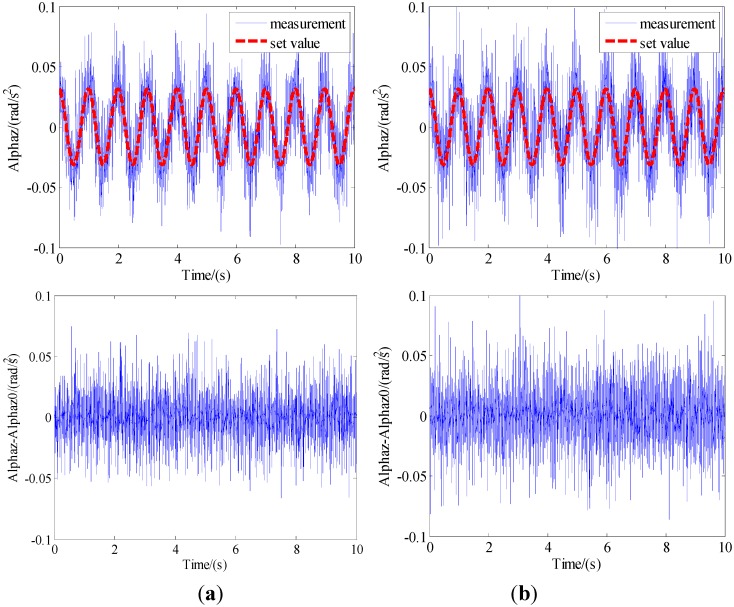
Comparison of measuring results of two configurations. (**a**) The proposed configuration; (**b**) The triangle planar configuration.

Then, experiments were carried out on a shaker. The linear accelerations were set as sinusoidal movement with 0.05 m/s^2^ amplitude and 1 Hz frequency and the angular velocity was set as a sinusoidal movement with 0.005 m/s amplitude and 1 Hz frequency. In the same time interval, the differences between the calculated angular accelerations and the set value for the two configurations are shown in Figure 11. During the same period, the RMS error of the difference of the angular acceleration between the measurements and the actual motion for the two configurations is shown in Table 2. Comparing to the planar triangle configuration, the RMS error of the angular acceleration has a reduction of 19.73%.

**Table 2:** Comparison of RMS error of angular acceleration differences.

RMS Error	The Proposed Configuration	The Triangle Planar Configuration
σ_α__x_	0.0119	0.0148
σ_α__y_	0.0116	0.0157
σ_α__z_	0.0118	0.0133
Error reduction	19.73%	0

**Figure 12:**
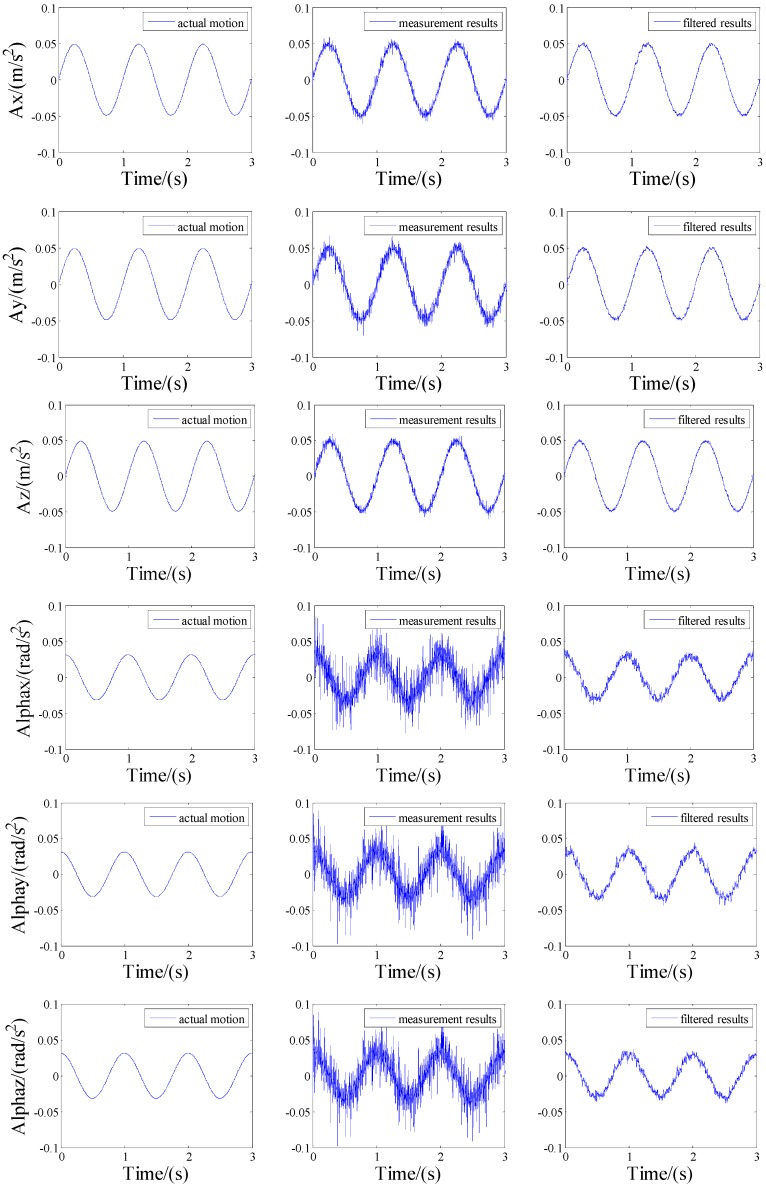
Comparison of true acceleration, measurement results and filtered results.

According to the prediction step and update step of the classical Kalman filter algorithm [19], the measurement results after filtering seen in Figure 12 can be obtained. The comparisons indicate that the angular acceleration formulae in the paper are correct, although there are errors due to the inaccurate positioning and orientation of the accelerometers, noises in the accelerometer outputs, and so on. Comparisons of RMS error between measurement results and filtered results are listed in Table 3. The RMS error can reduce 70.61% after filtering. All experiments prove that the configuration proposed in this paper has higher measurement precision.

**Table 3:** Comparison of RMS error between measurement results and filtered results.

RMS Error	Measurement Results	Filtered Results
αx	0.0117	0.0034
αy	0.0119	0.0034
αz	0.0118	0.0036
Error reduction	0	70.61%

## 5. Conclusions

A measurement model of accelerometers with high angular acceleration calculation precision was proposed for a maglev vibration isolation platform in this paper. Increasing the installation distance as much as possible within the effective space is an efficient method to minimize the angular acceleration RMS error. The influence of installation position and orientation errors of accelerometers on the proposed configuration and the previous planar triangle configuration were analyzed by simulation. Simulation results show that output values of the proposed configuration has higher measurement precision under the same installation error conditions. Experiments were conducted to compare the RMS noise of the angular accelerations of the proposed configuration and the planar triangle configuration. In a stationary environment, the RMS noise of angular acceleration of the proposed configuration has a 14.89% reduction comparing to the planar triangle configuration. During sinusoidal motion, the proposed configuration has a 19.73% reduction when comparing the differences of the RMS error of angular acceleration between the measurement results and the set value. By adopting the Kalman filtering method, the RMS error can be reduced by 70.61%. The experimental results prove that the proposed configuration has higher accuracy, and it is suitable for different platforms.
